# Outflow monitoring of a pneumatic ventricular assist device using external pressure sensors

**DOI:** 10.1186/s12938-016-0204-z

**Published:** 2016-08-25

**Authors:** Seong Min Kang, Keun Her, Seong Wook Choi

**Affiliations:** 1 Department of Mechanical and Biomedical Engineering, College of Engineering, Kangwon National University, 192-1 Hyoja-Dong, Chuncheon-si, South Korea; 2 Department of Cardiovascular and Thoracic Surgery, Soonchunhyang University Hospital, Bucheon-si, South Korea

## Abstract

**Background:**

In this study, a new algorithm was developed for estimating the pump outflow of a pneumatic ventricular assist device (p-VAD). The pump outflow estimation algorithm was derived from the ideal gas equation and determined the change in blood-sac volume of a p-VAD using two external pressure sensors.

**Objectives:**

Based on in vitro experiments, the algorithm was revised to consider the effects of structural compliance caused by volume changes in an implanted unit, an air driveline, and the pressure difference between the sensors and the implanted unit.

**Methods:**

In animal experiments, p-VADs were connected to the left ventricles and the descending aorta of three calves (70–100 kg). Their outflows were estimated using the new algorithm and compared to the results obtained using an ultrasonic blood flow meter (UBF) (TS-410, Transonic Systems Inc., Ithaca, NY, USA).

**Results:**

The estimated and measured values had a Pearson’s correlation coefficient of 0.864. The pressure sensors were installed at the external controller and connected to the air driveline on the same side as the external actuator, which made the sensors easy to manage.

## Background

A ventricular assist device (VAD) is effective for treatment of systolic end-stage heart failure, and can support the patient’s blood circulation until heart transplantation [1–8]. In fact, patients’ 1-year survival rates were three times higher when a VAD was used compared to drug therapies alone [6–8]. Despite the advantages of VADs, they are not widely implemented because of their high cost and risk of critical failure [6–9]. Malfunctions and inappropriate control of VADs can seriously damage organs and vessels; therefore, the outflow of VADs must be monitored to detect device failure or changes in the patient’s physiology [10–15].

The estimation of outflow using blood pressure sensors or motor speed has been studied; specifically, estimation according to the VAD outflow has been compared to that measured by an ultrasonic blood flow meter (UBF) [13–19]. The inlet blood pressure of the VAD has been shown to detect abnormal blood inflow; however, blood pressure sensors are difficult to manage for long periods because of their limited life span and difficulties in preventing thrombosis and fibrosis around the sensors [15, 16]. Outflow estimation has been studied based on the relationship between the motor speed and current consumption under normal hemodynamic conditions [17–20]. However, pathological changes in the patient’s physiologic state, or unexpected device failures, change the relationship between motor speed and current consumption. Although measurement with a UBF has been used to monitor the outflow of the VAD, the large size and complicated management process requires improvement [10, 13].

Pulsatile VADs (p-VAD) or artificial hearts may provide more effective measurements, since p-VADs pulsate blood in the implanted unit with transferred air expanding the air pocket [2, 8, 12, 21, 22]. The internal pressure can change the volume of the air pocket in the implanted unit; however, the air pressure cannot be used to calculate the volume change without considering the air moving to and from the implanted unit [22].

Measurement of the number of molecules of air with a VAD is not possible due to the structural deformation that the devices cause, as well as regulation of pulsation and suction force. Instead, the air through the airline can be measured using two air pressure sensors [21]. The change in volume of the air pocket in the implanted unit is calculated according to the amount of air molecules and the internal pressure in the implanted unit. In this study, a new algorithm for determining the stroke volume (SV) and outflow of p-VADs was developed by measuring the internal pressure and the number of air molecules in the external unit. The data obtained using this algorithm was verified by in vivo and in vitro experiments.

## Methods

### Design of the stroke volume estimation system of a p-VAD

**Fig. 1:**
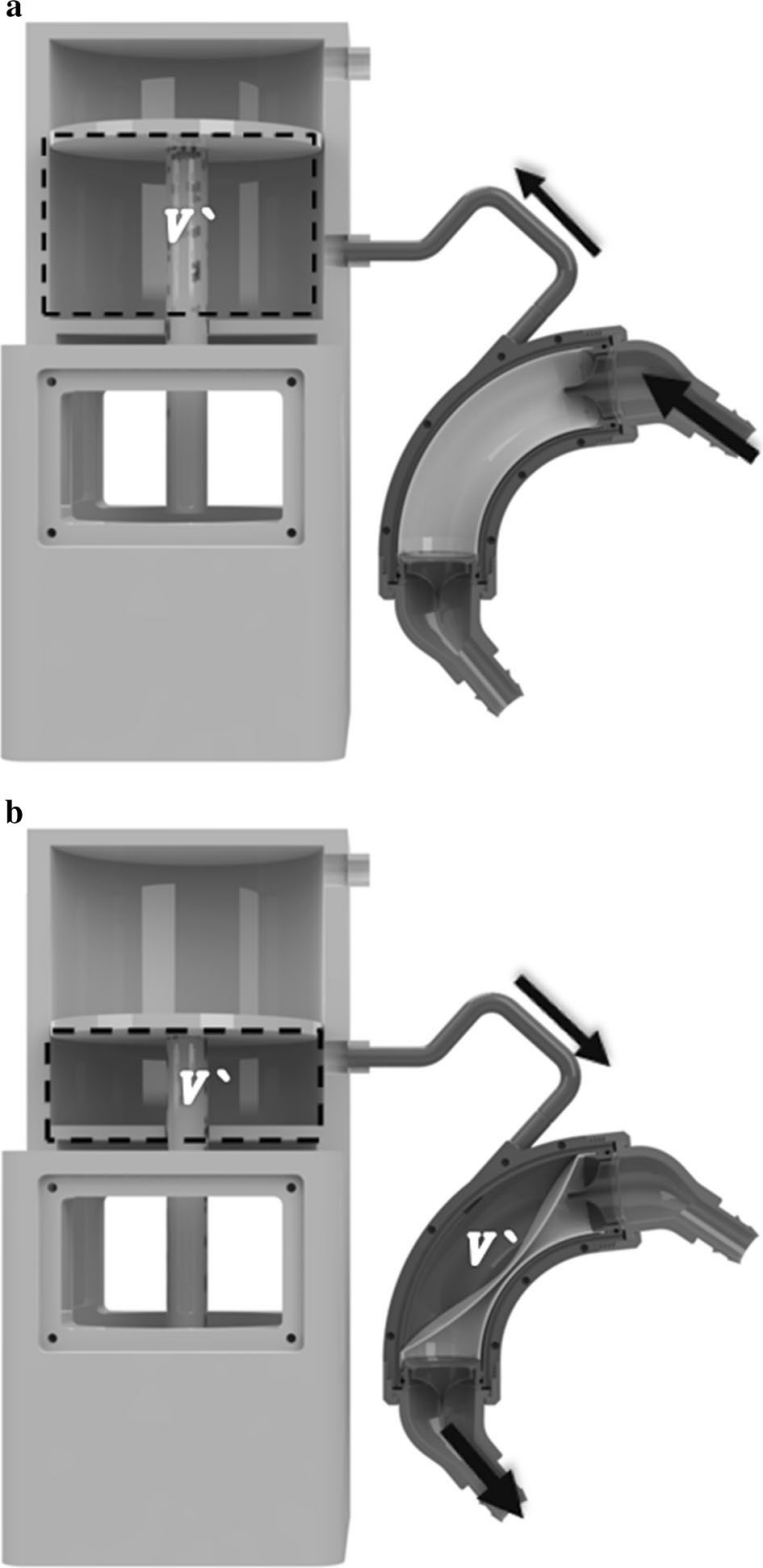
Variation of stroke volume (SV) according to air movement in the external driving unit. **a** Initial state volume (*V*’), **b** variation in volume according to air movement in cylinder (*V*’)

The SV of a pneumatic p-VAD is determined by the expanding volume of the air pocket in the unit, which is driven by an external pneumatic actuator (as shown in Fig. 1) [12]. However, there is a major difference between the expanding air volume of the implanted unit and the ejected air volume from the external actuator [21]. The air pressure of the implanted unit depends not only on the driving force from the external unit, but also on the inlet and outlet blood pressures of the implantable unit [22]. The inlet and outlet blood pressures could be changed according to the patient’s hemodynamic conditions. To detect the pump SV and the expanding volume of the air pocket in an implanted unit, data including the number of air molecules was considered in addition to the internal pressure of the implanted unit, according to an ideal gas equation.

**Fig. 2:**
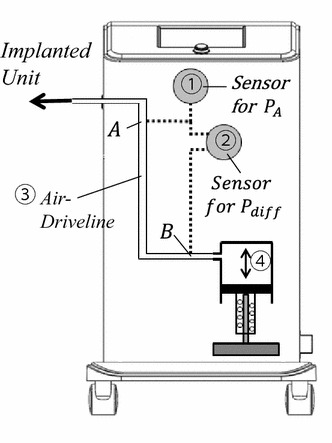
Configuration of the pump outflow estimation system of an external driving unit (① The mean pressure sensor, ② the differential pressure sensor (*A* to* B*), ③ the airline, and ④ the actuator)

Although the number of air molecules in the air pocket could not be determined by the internal pressure alone, the transfer of air molecules through the air driveline could be measured by the differential air pressure sensor. Differential pressure can also be useful to estimate the internal pressure in the implanted unit without the requirement of an additional sensor in the implanted unit, by considering the length of the air driveline. Therefore, the p-VAD system estimated SV and pump outflow with a differential pressure sensor (MPX2100AP; Freescale Semiconductor, Inc., Austin, TX, USA) and an absolute pressure sensor, which were embedded in the external actuator as shown in Fig. 2. The measuring points, A and B, of those pressure sensors were also located in the external actuator.

**Table 1:** Estimation parameters and measured

	(Unit)	Parameters for estimation	Measured parameters
R_AV_	(mmHg s/cc)	–	0.11 ± 0.01
R_AB_	(mmHg s/cc)	0.125 (Eq. 4)	0.15 ± 0.5
l_A–V_	(mm)	–	41 ± 0.5
l_A–B_	(mm)	–	201 ± 0.5
k_1_	(mol/mmHg_2_ s)	–	2.69 ± 0.05 (at 25 °C)
k_2_	–	5 (Eqs. 2, 3)	4.9 ± 0.05
C	(cc/mmHg)	0.21 (Eq. 3)	0.3 ± 0.01

In this study, the LibraHeart I (LibraHeart, Inc., Jeju, Korea) was used as a p-VAD with a tube-shaped blood sac that was designed to reduce the region of stationary blood to prevent thrombosis [12, 23]. Similar to a conventional p-VAD, the LibraHeart I has an external actuator and an implanted unit. The external actuator ejected air through the driveline. The volume of air transferred through the driveline was obtained by dividing the resistance between the two measured points by the differential pressure, according to Ohm’s law. However, because resistance according to air density, the amount of transferred air was determined using Eq. (1), which was derived from an ideal gas equation:

1 $$ [n_{A - B} ]_{0}^{t}   = k_{1} P_{A} (t)\mathop \int \nolimits_{0}^{t} P_{diff} (t)dt $$

$$ \left( {k_{1} = \frac{1}{{RR_{A - B} T}}, P_{diff} (t) = P_{A} (t) - P_{B} (t)} \right) $$

n_A−B_: mole number of passing air molecules at A

P_A_(t): pressure at A

P_B_(t): pressure at B

P_diff_(t): difference in pressure between A and B

R: gas constant (= 62.4 × 10^3^ cc mmHg/mol K)

k_1_: mole number—air pressure ratio (of air passing from A to B).

The air that passed through the driveline affected the air volume and pressure. Moreover, the driveline air changed the volume of the air pocket to that of the blood ejection or filling volume. To obtain the difference in air pocket volume, the internal pressure of the unit was measured along with the volume of transferred air. When the molecules of passing air were the same as that determined by Eq. (1), the internal pressure was measured using a differential pressure sensor and an absolute sensor according to Eq. (2).

2 $$ P_{V} (t) = P_{A} (t) - k_{2} P_{diff} (t) $$

$$ \left( {k_{2} = \frac{{l_{A - V} }}{{l_{A - B} }}} \right) $$

P_V_(t): pressure at the implanted unit of the VAD

l_A−V_: length from A to the implanted unit of the VAD (l_A−V_)

l_A−B_: length from A to B

k_2_: length ratio between l_A−V_ and l_A−B_.

The air pocket volume of the implanted unit was determined by the differential and absolute pressure set at A and B in Fig. 2. It is important to note that the internal pressure of the implanted unit and the air driveline expand those internal volume and their internal volume expansion decreases the effect of air pocket expansion on SV. Equation 3 suggests that the implanted unit is deformed and expanded by the internal pressure. The effective internal volume, V(t) was obtained with Eq. 4 to find the effect of both air pocket expansion and the expansion of structure space on the SV. The SV was determined as the difference between the maximum and minimum of the effective internal volume from Eq. 5. Table 1 shows the previously measured parameters and the used parameters for the estimation from the Eqs. 1 to 5.

3 $$ V'(t) = C\left( {\frac{{P_{A} (t) + P_{V} (t)}}{2} - P_{ext} } \right) = C\left( {\frac{{2P_{A} (t) - k_{2} P_{diff} (t)}}{2} - P_{ext} } \right) $$

$$ \left( {k_{2} = \frac{{l_{A - V} }}{{l_{A - B} }}} \right) $$

4 $$ \text{V}(t) = \frac{1}{{R_{A - B} }}\frac{{P_{A} \left( t \right)\mathop \smallint \nolimits_{0}^{t} P_{diff} \left( t \right)dt}}{{P_{V} \left( t \right)}} - V'(t) $$

5 $$ SV|_{pulse\_period} = [\hbox{max} \left( {V\left( t \right)} \right) - \hbox{min} (V(t))]_{t - pulse\_period}^{t} $$

### In vitro and In vivo experiments for verifying the stroke volume

**Fig. 3:**
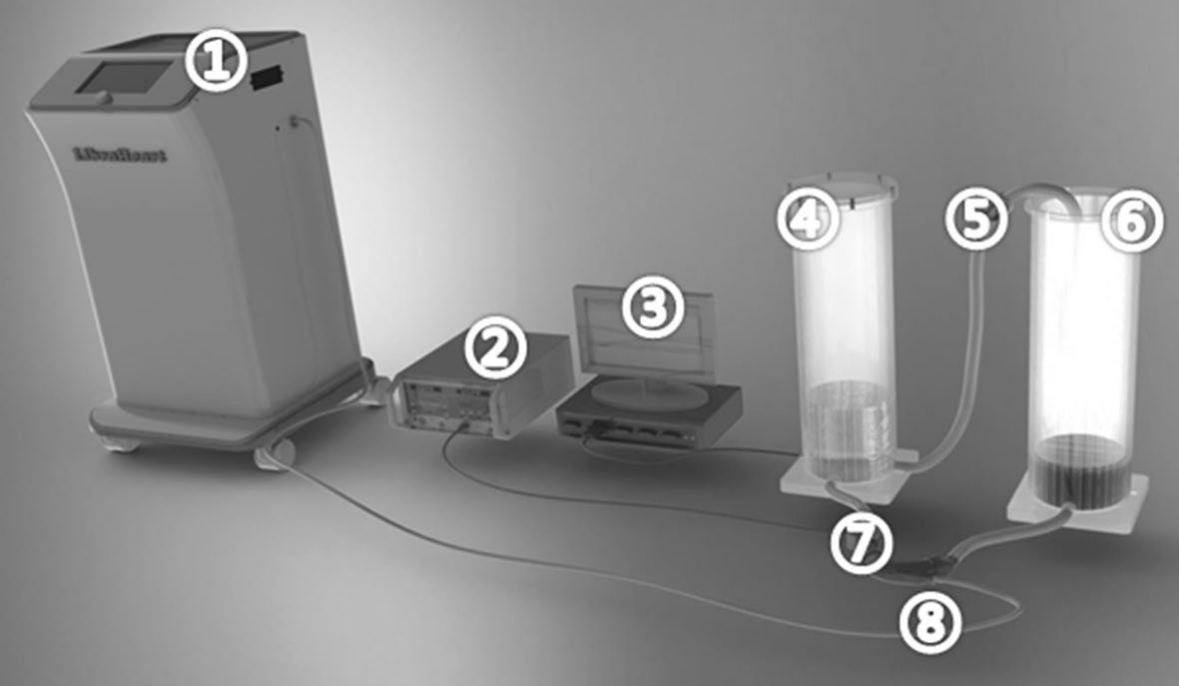
Mock circulatory system used to evaluate the pump outflow during the in vitro experiment. (① External driving unit, ② ultrasonic blood flow meter (UBF; TS-410, Transonic Systems Inc.) ③ pressure measurement device (MP36; Biopac Inc., Daegu, Korea), ④ outlet chamber used to simulate aortic pressure, ⑤ clamp to simulate the resistance of systemic perfusion, ⑥ the inlet chamber to simulate the left atrial pressure, ⑦ the UBF (PXL Clamp flow sensor; Transonic Systems Inc.), and ⑧ the implanted unit)

To evaluate the accuracy of the suggested SV estimation, a series of in vitro experiments were performed using a mock circulation system and compared to the results obtained using a UBF (Fig. 3). The probe of the UBF was positioned on the outlet tube of the implanted unit. A clamp was installed to adjust the blood pressure from 80 to 120 mmHg and, by adjusting the chamber, the peak-to-peak pressure changes were maintained at 40 mmHg. The SV was estimated from preloads ranging from 0 to −180 mmHg and afterloads ranging from 0 to 180 mmHg.

**Fig. 4:**
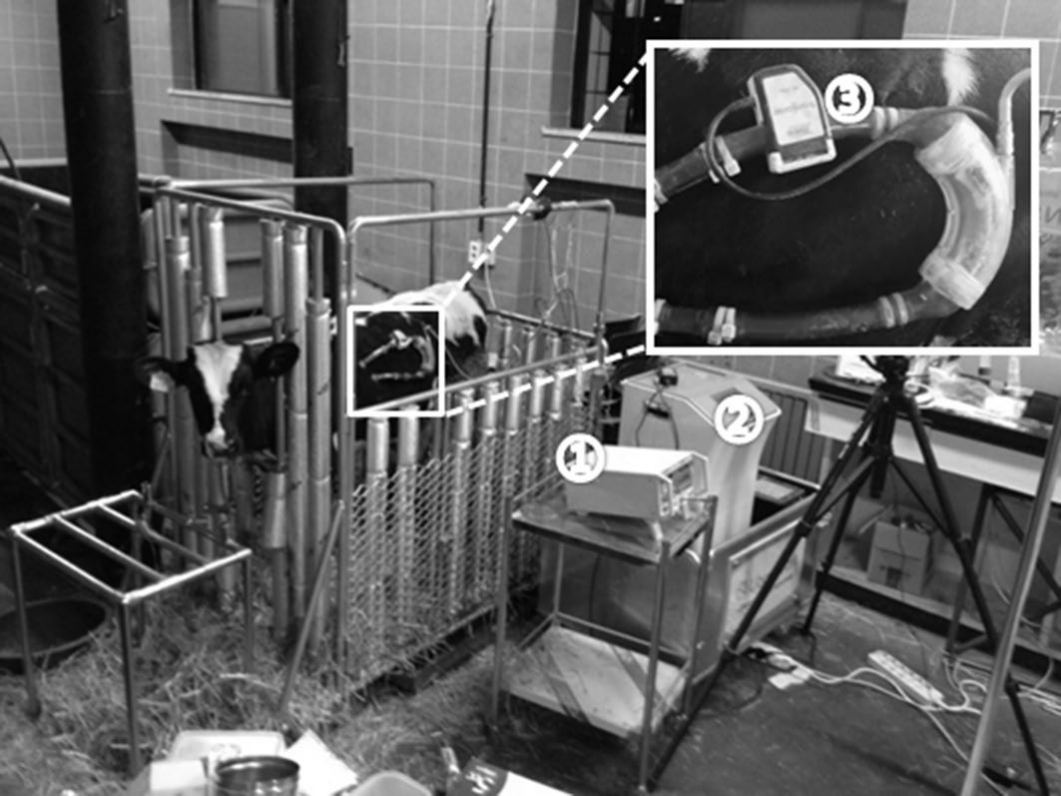
Short/long-term animal experiment (① the implanted unit, ② the ultrasonic sensor, ③ closed circuit television (CCTV), ④ the UBF, and ⑤ the external driving unit)

In this study, LibraHeart I p-VADs were connected to three calves, whose weights ranged from 76 to 98 kg, and the estimates of pump outflows were compared (Fig. 4). For connecting the left-ventricular assist device (LVAD), a thoracotomy was used to insert a catheter between the apex of the left ventricle and the descending aorta. For anesthesia, intramuscular ketamine (10 mg/kg) and inhaled isoflurane (1–2 %) were administered. Units were fixed to the animals’ backs and connected to the UFB probe for comparison to the estimated results. The inputs and outputs of implanted units were connected to the implanted catheter using 90 cm of Tygon tubing. The UBF probe was positioned on the cannula at the outlet side of the device. Following the operation, pump outflows for two animals were measured within 1 week due to failures in maintaining the flowmeter probe. The remaining animal was assessed after 3 months. The pump rate was set between 45 and 65 bpm and the SV of the p-VAD was maintained at 2–3 L/min.

## Results

**Fig. 5:**
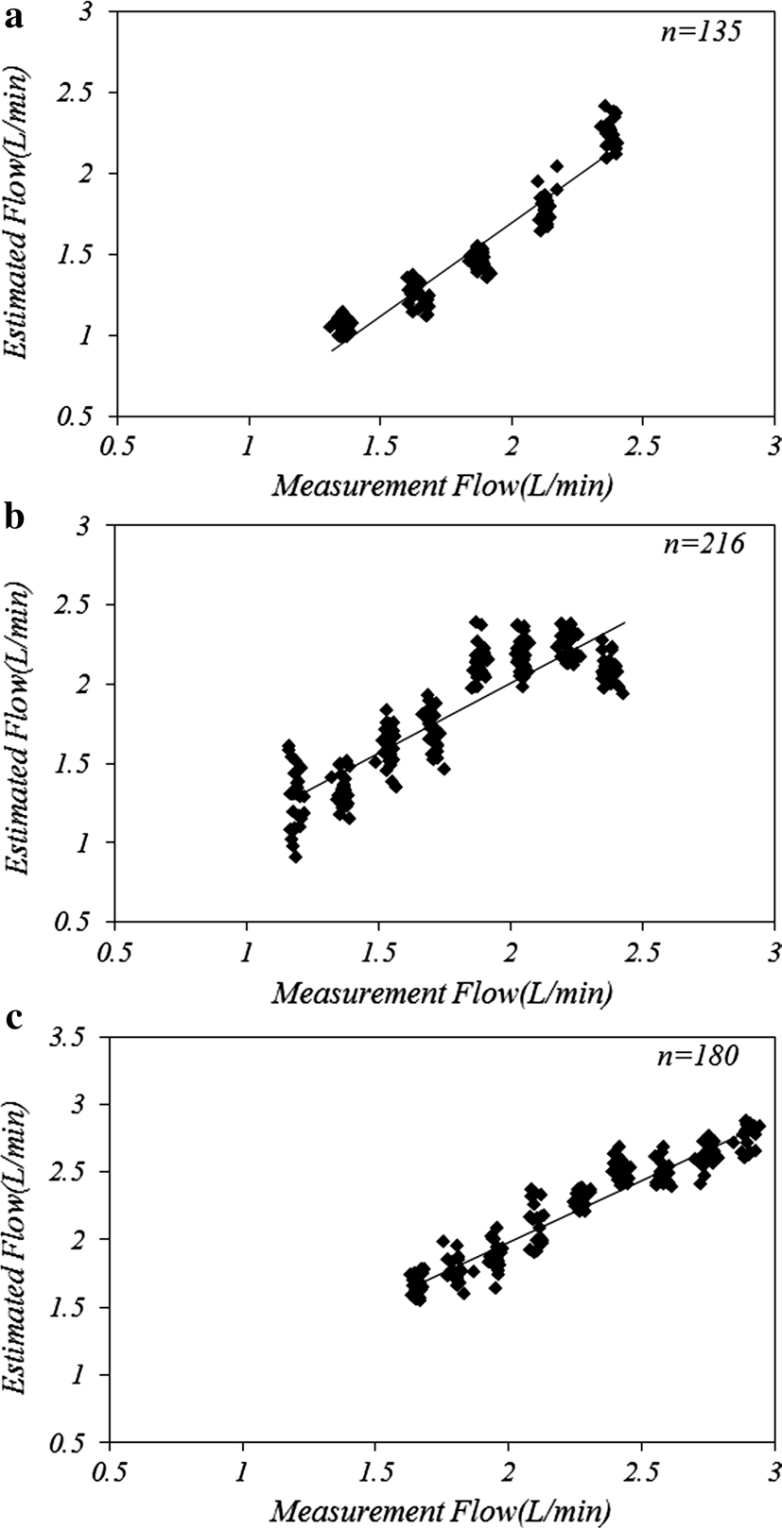
Correlation between the estimated and measured pump outflow. Correlations for **a** the in vitro preload, **b** in vitro afterload, and **c** in vitro bpm are shown

The results from the in vitro experiments are shown in Fig. 5. Figure 5a reveals a close relationship between the estimated SV and the volume measured by UBF. The preload was increased from 0 to −180 mmHg, with a reduction in the p-VAD outflow from 2.42 ± 0.23 to 1.15 ± 0.17 L/min. The Pearson’s correlation coefficient for the measured and estimated pump outflow was 0.966. Figure 5b shows the estimated and measured SV, based on the afterload. As the afterload was increased from 0 to 180 mmHg, the pump outflow was reduced from 2.41 ± 0.19 to 1.25 ± 0.27 L/min. When the afterload was changed over wide ranges, the Pearson’s correlation coefficient between the measured and estimated pump outflows was 0.88. Figure 5c shows the estimated and measured SVs according to the pump rate. Pump rate was changed from 40 to 80 bpm and the SV changed from 1.55 ± 0.17 to 2.81 ± 0.26 L/min.

**Fig. 6:**
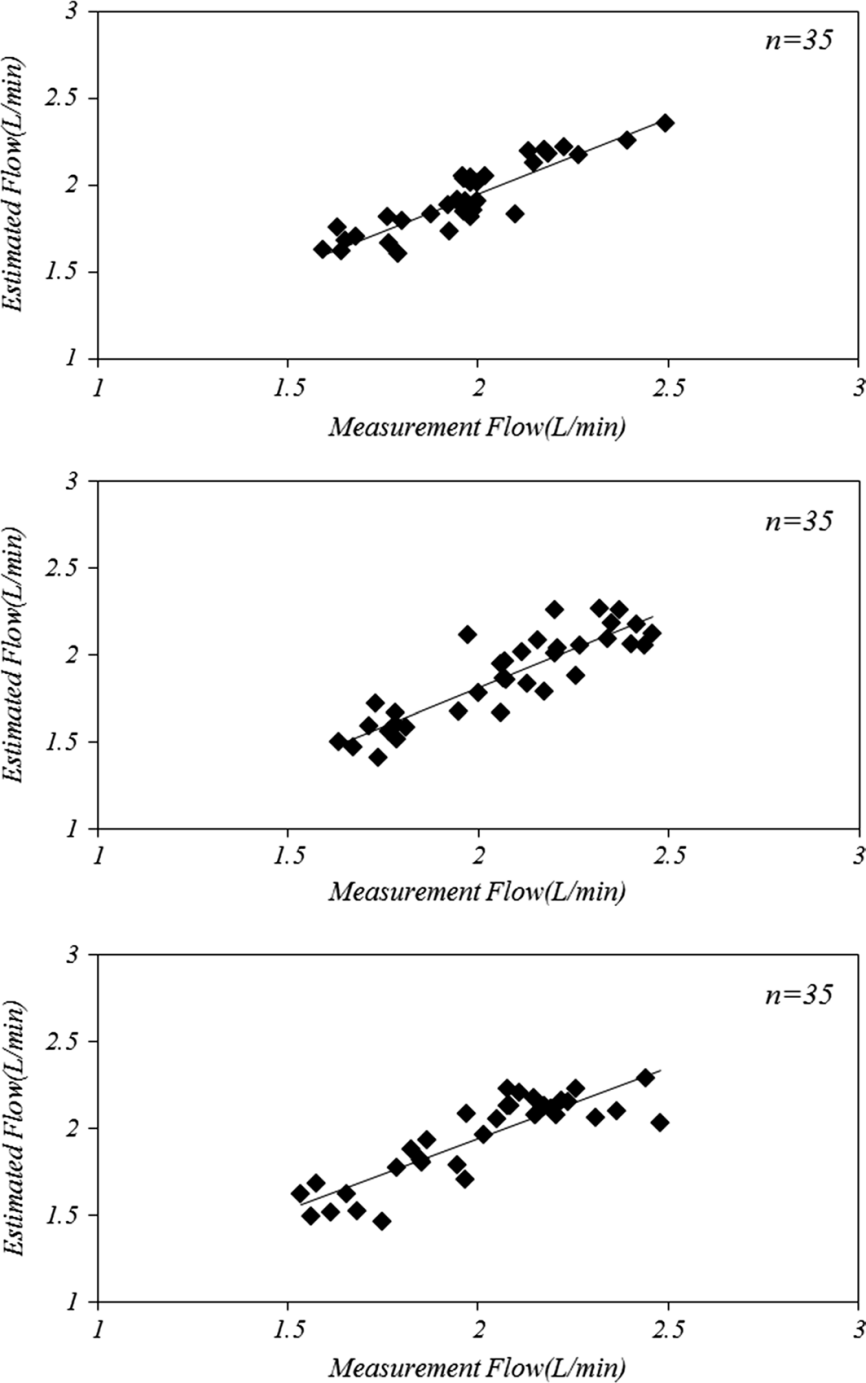
Correlation between the estimated and measured pump outflows, in vivo

The results of the in vivo experiment are shown in Fig. 6. A close relationship was observed between the measured and estimated pump outflows. To increase the preload and afterload, the inlet and outlet cannula on both sides of the device were clamped. The mean outflow of blood was maintained at 2.1 L/min until the preload and afterload were increased and the outflow was decreased to 0.6 L/min. For the three independent animal experiments, the Pearson’s correlation coefficients were 0.901, 0.878, and 0.867.

**Fig. 7:**
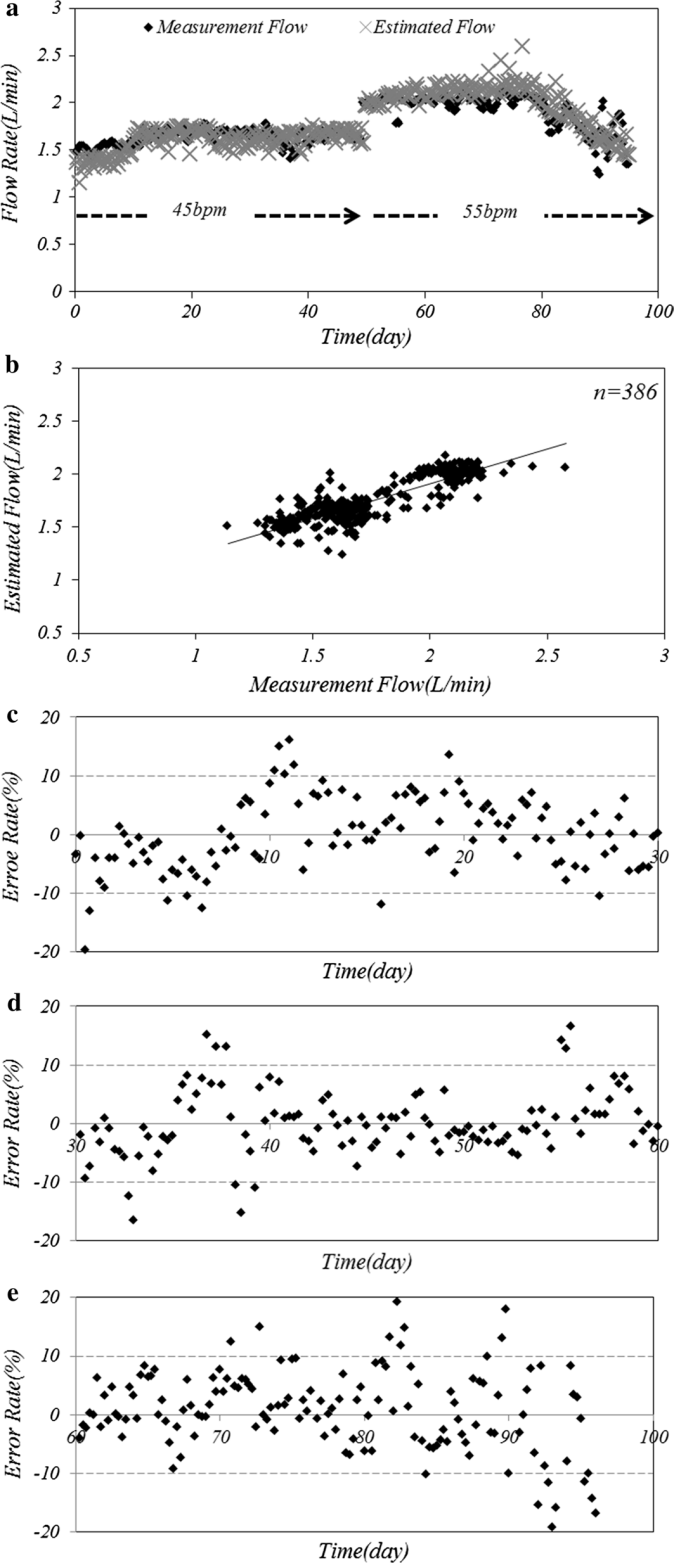
Long-term in vivo experiments shown are **a** variations between the estimated and measured pump outflow over 95 days, **b** the correlation between the estimated and measured pump outflow, **c** the error rate during early period from 0 to 30 days after the operation, **d** the error rate during middle period from 31 to 60 days and **e** the error rate during final period over 61 days

The results of the long term in vivo experiment are shown in Fig. 7. Figure 7a shows the estimated and measured SV during 95 days, Fig. 7b reveals a close relationship between the estimated SV and the measured volume using UBF and their Pearson’s correlation coefficient was 0.863. Figure 7c, d and e shows that the SV estimation did not changed in course of time.

## Discussion and conclusion

The estimation method suggested herein can be applied to conventional pneumatic p-VADs; however, it cannot be applied to the continuous rotary pumps that are primarily used at present [8]. The p-VAD is currently used for children or infants, where it has been applied to pneumatic artificial hearts [2, 24]. Moreover, the suggested method can also be applied to an intracortical balloon pump (IABP) to determine any expanding of the volume of the balloon. Thus, the technique can improve the safety of medical devices that are driven by air pockets or balloons [25].

The technique described herein estimates the inner pressure of the air pocket and considers the load on both of the implanted devices. The previously used UBF indicated blood flow only and could not be used to determine the cause of abnormal flow. Furthermore, while the current and motor speed could not be used to determine whether the abnormal findings were due to a failure of the device or hemodynamic changes in the patient, the estimation method presented herein determined that the device failed [19, 20]. In this study, errors caused by temperature changes were not observed. During in vitro experiments with a mock circulation system, the temperature of the mock system and the air pocket were between 21° and 25°. During the in vivo experiments, the implanted unit was attached to the animal’s back and the air tube was maintained at ambient temperature.

Within a closed chamber (EBE-4HW6P4C-20, ESPEC corp., Japan) of which internal temperature was adjusted from 5° to 40°, the correlation between the estimation SV and the measured was not changed. Even when the calculated effective volume V(t) in Eq. 4 was different according to temperature changes, the SV in Eq. 5 was not changed since the peak-to-peak value of V(t) waveform was not affected by temperature. Within this temperature range, the material compliance and structural volume were not changed and the temperature seemed to equally affect to the air volumes and pressures of the air driveline near sensors and the implanted unit. When the atmospheric pressure was decrease from 770 to 680 mmHg, the LVAD outflow slightly decreased and the SV estimation also decreased same to the measurement. If the temperature and pressure were out of the measuring range, the material compliance and structural space can change and cause the estimation error. The effects of severe temperature and pressure on material, structure and SV estimation will be studied with an advanced chamber.

In this study, the preload and afterload to the VAD affected the SV estimation differently. In our estimations, the compliance of air drive line and implanted case was set a value (0.05 cc/mmHg). However, the compliance could be different according to its internal air pressure. When the internal pressure was higher than atmospheric pressure, the compliance was maintained constant value, however, when the internal pressure was −100 mmHg lower than atmospheric pressure, the tube structure were deformed and its compliance got high compliance because of the buckling phenomenon and the abnormal bending curvature of the airline. The compliance of our structure was measured from 200 to −100 mmHg and the compliance was rarely changed within this range. In the in vitro experiments that applied preload below −30 mmHg to the LVAD, temporarily low internal air pressure below −100 mmHg frequently had been shown in the air drive line and implanted case. Those unexpected compliance increase caused the error of SV estimation results when the preload was applied to the VAD.

The LibraHeart I p-VAD used in this study contains a tube-shaped blood sac that reduces stagnation, which could otherwise cause thrombogenesis in the blood sac. Although the VAD has a blood sac that is different to that of other pneumatic p-VADs, it also contains an air pocket that is driven by an external actuator. Thus, the estimation method that was applied to the LibraHeart I in this study can also be applied to other devices. The method presented herein also facilitates management of the device, since the data were obtained using sensors located on the external actuator.
